# High efficacy of onabotulinumtoxinA treatment in patients with comorbid migraine and depression: a meta-analysis

**DOI:** 10.1186/s12967-021-02801-w

**Published:** 2021-03-31

**Authors:** Oreste Affatato, Thiago C. Moulin, Claudia Pisanu, Victoria S. Babasieva, Marco Russo, Elif I. Aydinlar, Paola Torelli, Vladimir N. Chubarev, Vadim V. Tarasov, Helgi B. Schiöth, Jessica Mwinyi

**Affiliations:** 1 Department of Neuroscience, University of Uppsala, Uppsala, Sweden; 2 Department of Biomedical Sciences, University of Cagliari, Cagliari, Italy; 3 Department of Pharmacology, Institute of Pharmacy, I. M. Sechenov First Moscow State Medical University, Moscow, Russia; 4 Neurology Unit, Neuromotor and Rehabilitation Department, Azienda USL-IRCCS of Reggio Emilia, Reggio Emilia, Italy; 5 Department of Neurology, Acibadem University School of Medicine, Istanbul, Turkey; 6 Headache Centre, Department of Medicine and Surgery, University of Parma, Parma, Italy; 7 Institute for Translational Medicine and Biothechnology, I. M. Sechenov First Moscow State Medical University, Moscow, Russia

**Keywords:** OnabotulinumtoxinA, Botox, Migraine, Depression, Meta-analysis

## Abstract

**Background:**

Migraine and depression are highly prevalent and partly overlapping disorders that cause strong limitations in daily life. Patients tend to respond poorly to the therapies available for these diseases. OnabotulinumtoxinA has been proven to be an effective treatment for both migraine and depression. While many studies have addressed the effect of onabotulinumtoxinA in migraine or depression separately, a growing body of evidence suggests beneficial effects also for patients comorbid with migraine and depression. The current meta-analysis systematically investigates to what extent onabotulinumtoxinA is efficient in migraineurs with depression.

**Methods:**

A systematic literature search was performed based on PubMed, Scopus and Web of Science from the earliest date till October $30{th}$, 2020. Mean, standard deviation (SD) and sample size have been used to evaluate improvement in depressive symptoms and migraine using random-effects empirical Bayes model.

**Results:**

Our search retrieved 259 studies, eight of which met the inclusion criteria. OnabotulinumtoxinA injections administered to patients with both chronic migraine and major depressive disorder led to mean reduction of $- \;8.94$ points (CI [$- \;10.04, - \;7.84$], $\hbox {p} < 0.01$) in the BDI scale, of $- \;5.90$ points (CI [$- \;9.92, - \;1.88$], $\hbox {p} < 0.01$) in the BDI-II scale and of $- \;6.19$ points (CI [$- \;9.52, - \;2.86$], $\hbox {p} < 0.01$) in the PHQ-9 scale, when evaluating depressive symptoms. In the case of the migraine-related symptoms, we found mean reductions of $- \;4.10$ (CI [$- \;7.31, - \;0.89$], $\hbox {p} = 0.01$) points in the HIT6 scale, $- \;32.05$ (CI [$- \;55.96, - \;8.14$], $\hbox {p} = 0.01$) in the MIDAS scale, $- \;1.7$ (CI [$- \;3.27, - \;0.13$], $\hbox {p} = 0.03$) points in the VAS scale and of $- \;6.27$ (CI [$- \;8.48, - \;4.07$], $\hbox {p} < 0.01$) migraine episodes per month. Comorbid patients showed slightly better improvements in BDI, HIT6 scores and migraine frequency compared to monomorbid patients. The latter group manifested better results in MIDAS and VAS scores.

**Conclusion:**

Treatment with onabotulinumtoxinA leads to a significant reduction of disease severity of both chronic migraine and major depressive disorder in patients comorbid with both diseases. Comparative analyses suggest an equivalent strong effect in monomorbid and comorbid patients, with beneficial effects specifically seen for certain migraine features.

## Background

Migraine and major depressive disorder (MDD) are two highly prevalent disorders worldwide, being a leading cause of significant limitations in life quality and disability. Despite several pharmacological and psychotherapeutic treatments available, patients tend to respond poorly, which can in turn worsen their health condition. Part of the burden is caused by the coexistence of these two disorders. Migraine has a large comorbidity spectrum which comprises many prevalent psychiatric disorders including depression, anxiety, different types of phobias and panic disorders [1, 2]. It is assumed that the relationship between migraine and depression is of bidirectional character, since migraine increases significantly the risk of depression and vice versa [3, 4].

One of the new and innovative therapeutic strategies for both migraine and depression is the treatment with onabotulinumtoxinA [5]. This toxin is produced by the bacterium *Clostridium botulinum* and it prevents the release of acetylcholine from nerve endings, thus leading to muscle paralysis until the nerve develops new endings to communicate with the muscles. OnabotulinumtoxinA is typically used to treat muscle contraction or spasms, hyperhidrosis from armpits, urinary incontinence in adults with multiple sclerosis and spinal cord injury. However, many studies showed that injections of this toxin can improve chronic migraine and that it can have a  beneficial effect on MDD [5, 6].

The underlying mechanism by which onabotulinumtoxinA leads to the observed beneficial effects in depression or migraine is not well understood. In the case of depression, one hypothesis is that the application of this drug inhibits the feedback of facial expressions to the brain, which can affect emotions positively or negatively [7]. Negative emotions cause contraction of the corrugator and procerus muscles in the forehead leading to frowning, which is suppressed by the use of onabotulinumtoxinA. This hypothesis is supported by observations that the use of onabotulinumtoxinA leads to an improvement of symptoms especially in individuals with a higher level of baseline agitation, i.e. increased psychomotor activity of the facial muscles [5].

As for depression the mechanisms underlying the beneficial effect of onabotulinumtoxinA in migraine are not fully elucidated either [8]. It has been hypothesized that the injection of this toxin in the trigeminally-innervated cranio-facial-cervical region inhibits the release of CGRP (a neuropeptide which is known to play an integral role in the pathophysiology of migraine) from peripheral nociceptive neurons. This interferes with transient receptor potential channels, thereby reducing neuronal hyperexcitability and peripheral and central sensitisation [9]. In this sense, it is relevant to note that pre-clinical experiments have proven the efficacy of onabotulinumtoxinA [10].

Many studies have been performed addressing the effectiveness of onabotulinumtoxinA on depression and migraine separately [5, 6, 11]. No study has systematically analyzed the overall average effect of onabotulinumtoxinA in patients presenting comorbid migraine and depression. This meta-analysis aims thus to assess the effect of treatment with onabotulinumtoxinA on patients with both chronic migraine and major depressive disorder and compare these results with the studies that addressed the effect of this drug on patients that have only one of these two conditions.

## Materials and methods

### Search criteria

A systematic search of the literature in accordance with the PRISMA guidelines was carried out using the databases PubMed, Web of Science and Scopus as search engines. The literature search has been performed independently by Oreste Affatato and Victoria Babasieva. Any disagreements were settled through discussion and under the supervision of Dr. Jessica Mwinyi.

All articles published in English before October $30{th}$, 2020 were considered. Searching with PubMed and using as search keywords “botulinum toxin migraine depression” or “botox depression migraine”, we detected $\hbox {n} = 42$ and $\hbox {n} = 28$ articles, respectively. Searching in Web of Science and using “onabotulinumtoxinA migraine depression” as search condition $\hbox {n} = 43$ articles have been found. The key words used in Scopus were “botulinum toxin migraine depression” and this way $\hbox {n} = 257$ studies were detected. Altogether, a total of 370 studies have been gathered.

**Fig. 1:**
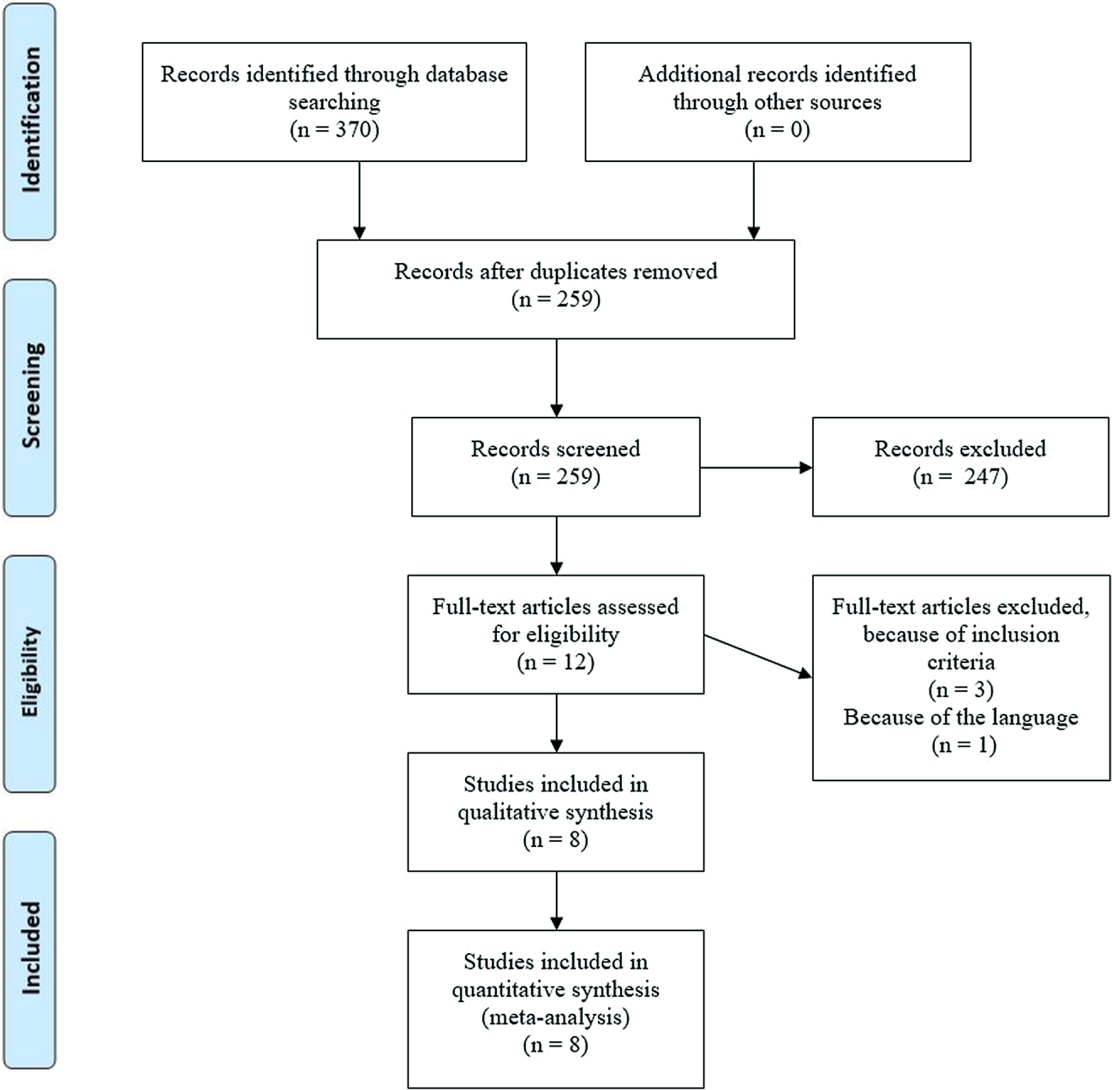
Preferred Reporting Items for Systematic Review and Meta-Analyses flow diagram [12] for the selection of the references

In the first screening of the articles, we considered only titles and abstracts. In this phase we excluded (i) articles not written in English, (ii) articles not reporting original data, such reviews, and (iii) articles not describing clinical experiments in which the aim was to assess the efficacy of onabotulinumtoxinA treatment in patients with both chronic migraine and major depressive disorder. At this stage, $\hbox {n} = 111$ articles were identified as duplicates and removed. From the remaining 259 studies, $\hbox {n} = 247$ records have been excluded because they were reviews or because those studies investigated subjects not related to our research. In a second screening we considered the full text of the articles. We included studies written in English and respecting the inclusion criteria, as stated below. The search and selection algorithm used in the study is summarized in Fig. 1.

### Inclusion criteria

Articles were selected on the basis of the following criteria: prospective or retrospective studies assessing the efficacy of the treatment with onabotulinumtoxinA in patients over the age of 18 years suffering from chronic migraine, as defined by any edition of the International Headache Society criteria [13], and from major depressive disorder, diagnosed by any scale.

### Statistical analysis

The studies selected follow the same protocol (PREEMPT) for the administration of the onabotulinumtoxinA [14], but they differed according to the scale used to assess impact and severity of migraine and depression, and regarding the duration of the treatment (from a minimum of 3 to a maximum of 27 months). For this reason, it was not possible to pool all the studies together for statistical analysis. Instead, studies were divided into subgroups according to the disease scales assessing the respective disorder and the duration of the treatment (3 subgroups for the depression, 3 subgroups for the migraine). It was possible to create a more comprehensive subgroup to assess the variation in the migraine frequency, i.e. the number of migraine attacks per month. A qualitative analysis of the studies considered in the meta-analysis is also provided to shed light on the treatment effect reported for each study.

From each study, the sample size as well as the mean decrease in migraine and depression scores from baseline including the standard deviation (SD) were extracted. In the few cases where mean reduction and SD were not reported, at least the means and the SD at the baseline and all the following visits during the treatment period were available. In these cases, data were used as follows. Let be $\mu _0$ and $\mu$ the mean values at the baseline and at a certain visit respectively, and $\sigma _0$ and $\sigma$ the associated SD. The mean reduction R was calculated using the formula

$$\begin{aligned} R = \mu - \mu _0 \end{aligned}$$

Since the distribution of the values is not given in the articles, it was not possible to calculate the covariance. Thus we calculated the SD associated with the mean reduction via the following (overestimating) approximation

$$\begin{aligned} \sigma _R = \sqrt{\sigma _0^2 + \sigma ^2} \end{aligned}$$

One study presented only the median, interquartile range (IQR) together with the sample size. In this case we followed the procedure published in [15] to give an approximation of the mean reduction in disease activity and SD.

The mean reduction and corresponding 95% confidence interval (CI) were derived using a random-effects model, which accounts for the intrinsic variation in the studies considered. The Empirical Bayesian method has been used to apply this model, known to be more accurate in the random-effects model when just few studies are under analysis [16]. It has been decided not to use a standardized effect size for two major reasons. First, many scales adopted in the various studies not only differ in the scoring system, but also in the epiphenomenon they evaluate, e.g. migraine severity and impact of migraine in the life of the patient. In this case, using a standardized effect size would lead to the comparison of effects that from a rigorous point of view could not be compared. An approach similar to ours has been used in [5, 17] and partly in [6, 11]. Second, from a clinical perspective, the standardized effect size has a less intuitive interpretation, while score reduction in a scale can easily lead to a clearer evaluation of the effect of the treatment.

To investigate the heterogeneity between studies Q and $\hbox {I}^2$ statistics were performed. $\hbox {p} \le 0.1$ was considered as a threshold for significant heterogeneity. $\hbox {I}^2$ values of 25, 50 and 75% were considered low, medium and high heterogeneity respectively.

It was not possible to assess the publication bias via Begg’s funnel plot, or correcting through Egger’s regression asymmetry test, due to the small number of studies available. We contacted all the authors of the studies included in the meta-analysis in order to ask for collaboration and sharing of possibly not published data. Doctor Elif Ilgaz Aydinlar [18] and doctor Marco Russo [19] shared all the data produced in their respective studies.

All p-values below 0.05 were considered as statistically significant.

The statistical analysis was carried out using the software Stata, version 16 (Stata Corp, College Station, TX, USA).

## Results

Based on the described inclusion criteria and the selection process presented in Fig. 1, eight studies were eventually selected for the analyses. The main epidemiological features of the selected studies are listed in Table 1. In all the studies the PREEMPT protocol [14] for the injection of the onabolutinumtoxinA was followed.

**Table 1:** Summary of the key epidemiological characteristics of the studies selected

Reference	Country	Total patients (% of women)	Mean age (SD)	Scale assessing depression	Scale assessing migraine
Maasumi et al. [20]	USA	359 (86.1%)	45.1 (13.2)	PHQ-9	HIT6
Boudreau et al. [21]	Canada	32 (87.5%)	42.4 (12.4)	PHQ-9, BDI-II	HIT6, MIDAS, VAS
Kollewe et al. [22]	Germany	27 (92.3%)	45.6 (10.8)	BDI	HIT6
Russo et al. [19]	Italy	52 (88.5%)	48.7 (12.9)	BDI-II	MIDAS
Demiryurek et al. [23]	Turkey	60 (73.3%)	34.7 (6.4)	BDI	MIDAS, VAS
Guerzoni et al. [24]	Italy	57 (80.7%)	54.2 (13)	Zung-D	HIT6, VAS
Aydinlar et al. [18]	Turkey	190 (87.9%)	39.3 (10.2)	DASS-21	MIDAS
Blumenfeld et al. [25]	USA	715 (84.8%)	43.0 (11.3)	PHQ-9	None

### Qualitative analysis

Maasumi et al. [20] performed a retrospective data analysis on chronic migraineurs that received more than 2 consecutive injections of onabotulinumtoxinA for 6-12 months. Migraine intensity was assessed by the Headache Impact Test (HIT6), while depression was evaluated by Patient Health Questionnaire-9 (PHQ-9). A total of 359 patients underwent the treatment (86.1% women, 92.8% Caucasian). They found that 108 (30.1%) patients improved significantly ($\hbox {p} < 0.0001$) in their HIT6 score by 6 or more points and among these patients, 41 (38%) subjects also improved significantly in the PHQ-9 score by 5 or more points. Among the patients who did not report a meaningful improvement in the HIT6 score, just 24/251 (9.6%) improved significantly ($\hbox {p} < 0.0001$) in the PHQ-9 score by 5 or more points.

Boudreau et al. [21] performed a prospective, open-label, multicenter pilot study on chronic migraineurs with associated depressive symptoms. The impact of migraine was assessed by the HIT6 and the Migraine Disability Assessment (MIDAS), while depression through PHQ-9 and the Beck Depression Inventory II (BDI-II). Anxiety was assessed as well via Generalized Anxiety Disorder-7 (GAD-7). Thirty-two patients  received the treatment (87.5% women, 78.1% Caucasian) for 6 months. After 6 months, they found significant improvement ($\hbox {p} < 0.0001$) in the number of headache/migraine-free days (mean + 8.2, SD 5.8). There were also a significant ($\hbox {p} < 0.0001$) improvement in the HIT6 score (mean $- \;6.3$, SD 6.9), in MIDAS score (mean $- \;44.2$, SD 67.5, $\hbox {p} = 0.0058$) as well as in the Visual Analogue Scale (VAS) score (mean $- \;2.5$, SD 2.5). Significant improvements (both with p $< 0.0001$) were measured with BDI-II (mean $- \;7.9$, SD 6.0) and PHQ-9 (mean $- \;4.3$, SD 4.7) compared to the baseline, after 6 months. Also the GAD-7 score improved compared to baseline (mean $- \;3.5$, SD 5.0, $\hbox {p} = 0.0002$). No adverse events were reported.

Kollewe et al. [22] performed a prospective observational study. Twenty-seven chronic migraineurs (92.3% women) received four consecutive onabotulinumtoxinA injections every 3 months. Migraine impact was assessed by HIT6 and depression severity by BDI. The researchers found a significant improvement ($\hbox {p} < 0.001$) in HIT6 score (mean $- \;19.4$, SD 5.6) and in BDI score (mean $- \;9.0$, SD 7.9). Monthly headache days were significantly ($\hbox {p} < 0.001$) reduced from a mean of 18.9 (SD 3.9) to a mean of 8.7 (SD 4.5). Adverse events were minor and transient.

Russo et al. [19] performed a prospective observational study. Fifty-two subjects affected by chronic migraine and depressive symptoms underwent the treatment for 15 months. Twenty-eight subjects had discontinuous treatment due to poor compliance (50%), inefficacy (35.7%) or poor tolerability (14.3%). After 6 months of treatment, a reduction ($\hbox {p} = 0.09$) in the MIDAS score was observed: from a mean of 71.7 (SD 43.4) to a mean of 51.9 (SD 65.5). Furthermore, a non-significant ($\hbox {p} = 0.12$) improvement in the BDI-II score was observed, from a mean of 17.9 (SD 10.1) to a mean of 14.1 (SD 11.7). A significant ($\hbox {p} = 0.002$) median reduction of $- \;2$ (IQR [$- \;7, 0$]) days of headache per month was also observed. After 9 months it has been observed a non-significant ($\hbox {p} = 0.21$) decrease in the MIDAS score: from a mean of 51 (SD 17.4) to a mean of 30.3 (SD 29). Similarly, a non-significant ($\hbox {p} = 0.40$) decrease in the BDI-II score was reported: from a mean of 15.7 (SD 7.8) to a mean of 14.1 (SD 11.2). Also in this case a significant ($\hbox {p} = 0.011$) median reduction of $- \;3.5$ (IQR [$- \;4.8, - \;1.5$]) days of headache per month was observed.

Demiryurek et al. [23] performed a prospective observational study in which the disability assessment of the chronic migraine was done through MIDAS test, while the depression was assessed via BDI. 60 adults (73.3% women) aged between 20 and 50 years old obtained two injections of onabotulinumtoxinA. No significant side effects were observed. MIDAS scores were significantly ($\hbox {p} < 0.001$) lower after the treatment, decreasing from a mean of 17.40 (SD 4.92) to a mean of 8.22 (SD 5.29). Likewise, the VAS score decreased from a mean of 8.90 (SD 0.75) to 6.53 (SD 1.44). It has been reported also a significant ($\hbox {p} < 0.01$) decrease in number of days with headache in a month, from a mean of 18.78 (SD 2.06) to a mean of 5.80 (SD 4.17). Significant ($\hbox {p} < 0.041$) decrease was also observed in BDI scores, from a mean of 16.13 (SD 9.29) to a mean of 7.67 (SD 4.63).

Guerzoni et al. [24] performed a retrospective study in a sample of 66 patients (90.7% women) with a diagnosis of chronic migraine associated with medication overuse, according to the International Classification of Headache Disorders (ICHD-III, beta). Depression severity was assessed by the Zung Self-Rating Depression scale (Zung-D), while migraine impact was measured by HIT6 scale. Just 57 patients had regular injections of onabotulinumtoxinA every three months without interruption up to seven cycles. 58% of the patients did not report any adverse event. Overall, no serious event was reported. In general, a significant ($\hbox {p} < 0.01$) decrease in the HIT6 scores was observed, from a mean of 63.94 (SD 6.91) at the beginning to a mean of 52.28 (SD 8.69) after completing the treatment. A significant ($\hbox {p} < 0.01$) reduction in the VAS score, from a baseline of 7.98 (SD 1.26) to a mean of 4.25 (SD 1.48) and a significant ($\hbox {p} < 0.0001$) reduction in the fraction of headache days per month, from a mean of 0.98 (SD 0.09) to a mean of 0.65 (SD 0.36) were reported. No significant decreases were measured with the Zung-D score.

Aydinlar et al. [18] performed a single-center prospective cohort study in which 190 patients (87.9% women) were recruited. Among these, just 10.5% of the patients completed all the planned cycles of injections. The therapy was associated with minor and temporary side effects. Migraine impact was assessed via the MIDAS score, while depression via the Depression Anxiety Stress Scale (DASS-21). Every cycle of injection was associated with significant decreases in headache frequency ($\hbox {p} < 0.001$) and severity (up to visit 4 $\hbox {p} < 0.001$, at visit 5 $\hbox {p} = 0.017$). Least squares mean MIDAS score decreased significantly from 67.3 at baseline (66 patients) to 17.4 at visit 2 (66 patients, $\hbox {p} = 0.001$), to 15.3 at visit 3 (47 patients, $\hbox {p} < 0.001$), to 9.3 at visit 4 (24 patients, $\hbox {p} < 0.001$) and to 18.5 at visit 5 (17 patients, $\hbox {p} < 0.001$). No significant changes were reported for the DASS-21 score.

Blumenfeld et al. [25] performed a multicenter, open-label, prospective study over 27 months. A total of 715 patients (84.8% women) were recruited and received at least one dose of onabotulinumtoxinA, but just 373 (52.1%) patients completed the study. The treatment was generally well-tolerated. Depression and anxiety levels were assessed via PHQ-9 and GAD-7 scales respectively.  Any improvement of at least one severity category (e.g. from moderate to mild) was considered clinically meaningful. A statistically significant mean reduction in headache days from a baseline of 22.0 (SD 4.8) days/month was observed at the sixth month (mean $- \;7.4$ days/month, SD 6.2) and sustained until the end of the study (mean $- \;10.7$ days/month, SD 6.4). Significant improvement has been reported also for the PHQ-9 and GAD-7 scores. By the end of the study, 53.4% of the patients who completed the study had a clinically meaningful improvement in depressive symptoms and 37.3% in anxiety symptoms.

### Results of quantitative analyses on major depressive disorder

**Fig. 2:**
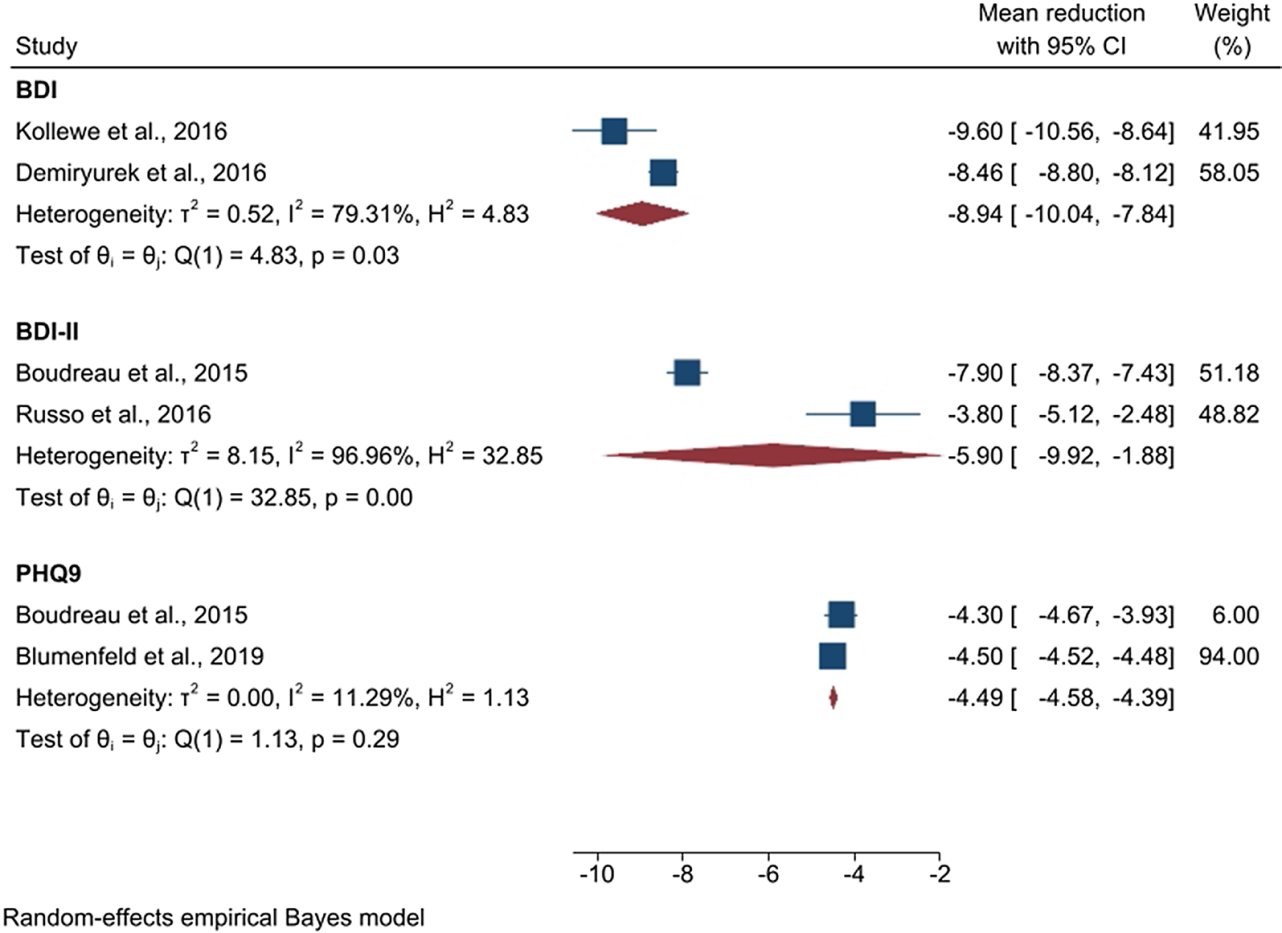
Forest plot of the subgroup analysis assessing the reduction in the depressive symptoms. We reported the mean reduction in BDI scores after 3 months of treatment and in BDI-II and PHQ-9 scores after 6 months of treatment

In the case of MDD, a subgroup analysis according to three different disease severity scales was performed, i.e. BDI, BDI-II and PHQ-9. Within each subgroup, the studies also presented different durations of treatment. For this reason, for each subgroup we selected the outcome data for the statistical analysis according to the shortest study. In some studies, the results at each visit along the treatment were not available. In these cases, we chose the data for the calculations according to the visit with more outcome variables available. This procedure was adopted to maximize the number of studies in each subgroup analysis. In the case of studies using the BDI scale, we considered the outcome results of the treatment with onabotulinumtoxinA after 3 months. In the case of studies operating with the scales BDI-II and PHQ-9 our calculations were performed based on treatment outcome data obtained 6 months after the treatment start. Meta-analysis results are presented in Fig. 2.

The BDI scale has been used to assess the depression level in a total of 87 patients. We found a significant (p $< 0.01$) mean reduction in scores of $- \;8.94$ (CI [$- \;10.04, - \;7.84$], $\hbox {z} = - \;15.89$) points. The BDI-II scale has been used in a total of 48 patients, while the PHQ-9 scale in case of 445 subjects. Significant ($\hbox {p} < 0.01$) mean reductions of $- \;5.90$ (CI [$- \;9.92, - \;1.88$], $\hbox {z} = - \;2.88$) and of $- \;4.49$ (CI [$- \;4.58, - \;4.39$], $\hbox {z} = - \;94.51$) points were seen for BDI-II and PHQ-9 scores, respectively.

While significant heterogeneity was detected for the results obtained based on the BDI and BDI-II scores, non-significant results for heterogeneity were obtained with PHQ-9. Due to the small number of studies included in each of the analyses, heterogeneity tests cannot be evaluated as reliable in the presented cases.

### Results of quantitative analyses on chronic migraine

**Fig. 3:**
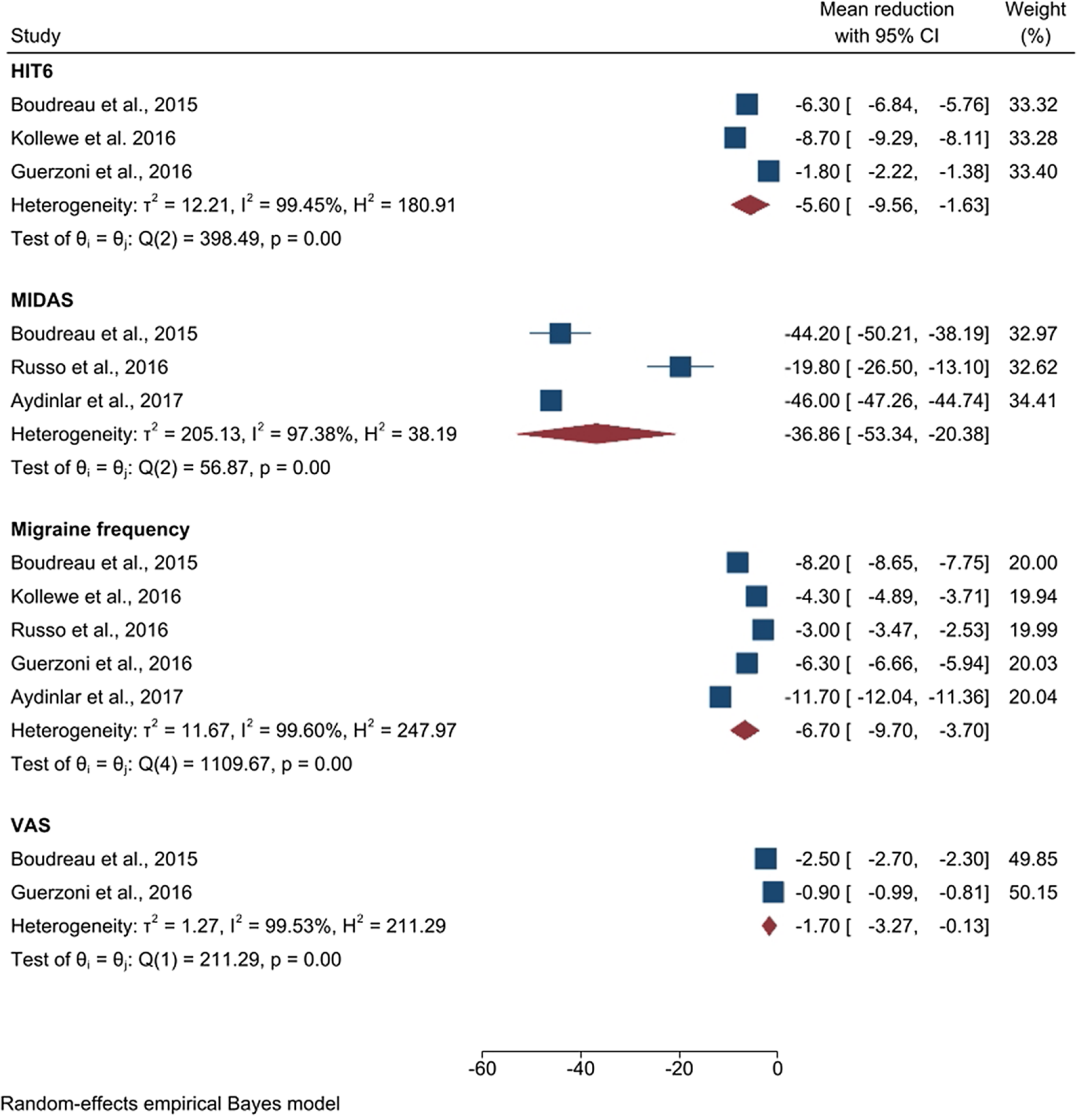
Forest plot of the subgroup analysis assessing the reduction in the migraine symptoms. We reported the mean reduction in HIT6, MIDAS, VAS scores and the mean reduction in migraine frequency (i.e. number of headache attacks per month) after 6 months of treatment

In the case of chronic migraine, a subgroup analysis has been performed for outcomes obtained with three scales, i.e. HIT6, MIDAS and VAS. We also conducted a broader analysis assessing the mean reduction in migraine frequency, i.e. the number of headache days per month. In all the cases, the calculations have been conducted using outcomes after 6 months of treatment. The results are shown in Fig. 3.

The HIT6 scale has been used to assess migraine severity level in a total of 102 patients. A significant result ($\hbox {p} = 0.01$) was observed detecting a mean reduction of $- \;5.60$ (CI [$- \;9.56, - \;1.63$], $\hbox {z} = - \;2.77$) points. The MIDAS scale has been used in a total of 92 patients, while the VAS scale was applied in 75 subjects. We obtained significant ($\hbox {p} < 0.01$) mean reductions of $- \;36.86$ (CI [$- \;53.34, - \;20.38$], $\hbox {z} = - \;4.38$) and of $- \;1.7$ (CI [$- \;3.27, - \;0.13$], $\hbox {z} = - \;2.12$, $\hbox {p} = 0.03$) points respectively. Data about mean reduction in migraine frequency have been extracted from 5 studies, involving a total of 180 subjects. A significant ($\hbox {p} < 0.01$) mean reduction of $- \;6.70$ (CI [$- \;9.70, - \;3.70$], $\hbox {z} = - \;4.38$) in migraine days per month was detected.

As in the case of the analysis on depression, the test for the heterogeneity cannot be considered reliable due to the small number of studies selected for this meta-analysis.

### Comparison between comorbid and non-comorbid patients

**Fig. 4:**
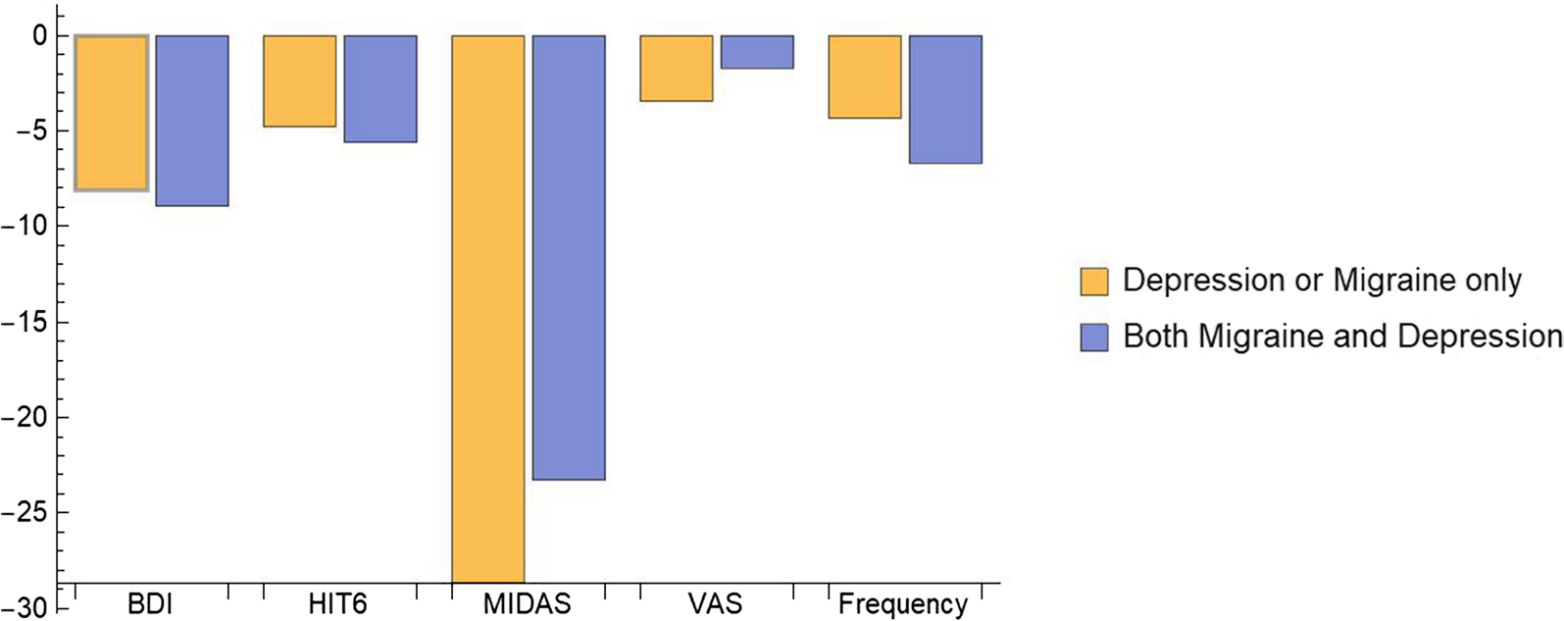
Comparison of the treatment efficacy with onabotulinumtoxinA in patients monomorbid with migraine or depression and patients co-morbid with both disorders. Data of the patients showing only migraine or depression were extracted from the meta-analyses [5, 6, 11, 17]. Data of comorbid patients were calculated based on the studies [18–25]. Comparisons were conducted on patients who have been treated with onabotulinumtoxinA over 3 (BDI and MIDAS) or 6 months (HIT6, VAS and migraine frequency)

Figure 4 shows the results from our meta-analysis studying the effect of onabotulinumtoxinA in patients with migraine and depression in comparison with the data extracted from other meta-analyses studying the overall effect of onabotulinumtoxinA in patients with just chronic migraine [6, 11] or just major depressive disorder [5, 17]. Due to several different scales used to assess depression throughout the publications, the only comparison possible to perform was based on results obtained with the BDI scale. The treatment outcomes reported after two months from the start were available from studies assessing just depression and after three months from studies assessing both migraine and depression. In the case of migraine, we reported the outcomes after three months of treatment for the MIDAS scale and after six months in all other cases. Results are calculated as mean reduction and 95% CI in those cases where data for such calculations were available.

Comparing outcomes obtained with the BDI scale, patients ($\hbox {n} = 55$) affected only by depression reported a mean reduction of $- \;8.12$ points, which was only to a minor extent lower compared to patients with migraine-depression ($\hbox {n} = 87$), those showing a mean reduction of $- \;8.94$ (CI [$- \;10.04, - \;7.84$]) points. The HIT6 has been used in a total of 688 chronic migraineurs, reporting a mean reduction of $- \;4.8$ (CI [$- \;5.00, - \;4.60$]) points, while 102 comorbid patients reported a mean reduction of $- \;5.6$ (CI [$- \;9.56, - \;1.63$]) points. Thus, a slightly better outcome for migraine is seen in comorbid patients compared to patients having only migraine based on the HIT6 scale.

The impact of the chronic migraine was assessed by using the MIDAS scale in a total of 48 migraineurs and in 149 subjects with both migraine and depression. A mean reduction of $- \;28.65$ points was detected in the first case, which was clearly higher compared to the reduction of $- \;23.29$ (CI [$- \;46.41, - \;0.16$]) points seen in the second case.

Using the VAS scale, 20 chronic migraineurs reported a mean reduction of $- \;3.45$ points, while 75 patients with comorbid migraine and depression reported a slightly lower mean reduction of $- \;1.7$ (CI [$- \;3.27, - \;0.13$]) points.

A total amount of 804 chronic migraineurs reported a mean reduction in migraine frequency of $- \;4.37$ (CI [$- \;9.05, 0.31$]), while 180 comorbid patients reported a clearly higher mean reduction of $- \;6.70$ (CI [$- \;9.70, - \;3.70$]).

## Discussion

To our knowledge, this is the first meta-analysis that assessed the efficacy of the onabotulinumtoxinA treatment administered to patients with comorbid chronic migraine and major depressive disorder. Previous meta-analytic studies addressed just one of the two disorders (i.e. [5, 17] for the depression and [6, 11] for the migraine). Another important feature of our research is the analysis of a broader set of clinical experiments on depression, as compared with previous meta-analyses on the same subject that included only three studies [5, 17]. Our results demonstrate that the  treatment with onabotulinumtoxinA leads to a significant improvement of both migraine and depressive symptoms in comorbid patients.

Migraine and depression are tightly connected disorders, as one increases significantly the risk to manifest the other. This close relationship is validated by common features in the pathophysiology and genetic predisposition of patients that manifest both disorders. The most probable mechanisms underlying the common pathogenesis are considered serotonergic dysfunction, ovarian hormone influences and dysregulation in the hypothalamic-pituitary-adrenal axis, along with a common genetic predisposition [26]. Based on results obtained in MRI analyses it has been proposed that comorbid migraine and depression may represent a pathology by itself, disjoint from migraine and depression separately [4]. More studies are needed to confirm this hypothesis. Nevertheless, this possibility urges the necessity to assess  to what extent the treatments used to cure migraine and depression separately  have a beneficial effect on patients with comorbidity.

Our meta-analysis demonstrated an overall statistically significant improvement of depressive symptoms and quality of life, as measured through PHQ-9, BDI and BDI-II scales, in patients with chronic migraine and MDD. Likewise, significant improvements in migraine severity and impact, as assessed by MIDAS, HIT6 and VAS scales were detected in the comorbid patient group. This is furthermore reflected in a considerable mean reduction in the migraine frequency. The measured improvements are not only meaningful from a meta-analytic perspective, since each study reported statistically significant improvements, pointing separately to the conclusion of the effectiveness of the onabotulinumtoxinA as a treatment for migraine and depression. These results confirm that the use of onabotulinumtoxinA in the comorbid patient group is an efficient alternative to treat both diseases as hitherto several times shown for the isolated conditions (i.e. [27–29] for depression, [30–34] for migraine) and confirmed through meta-analytic approach studying both conditions separately [5, 17, 6, 11].

Interestingly, onabotulinumtoxinA seems to enfold a slightly different beneficial effect dependent on whether a patient shows just one or both diseases together. As shown in Fig. 4, a slightly better outcome is seen for depression based on BDI scale in patients with both depression and migraine [5, 17] compared to patients showing depression only. In case of chronic migraine, the situation appears to be slightly more heterogeneous. Patients with just migraine manifested a clearer improvement in the MIDAS and VAS scales, compared with migraineurs with depression [6, 11]. On the other hand, and on the basis of even larger sample sizes, patients with both chronic migraine and depression reported a greater improvement in their conditions, as assessed by the HIT6 scales and by the migraine frequency, compared with patients who showed chronic migraine only.

Current data suggest that onabotulinumtoxinA shows an at least comparable efficacy on depression and migraine in comorbid and monomorbid patients, based on observational time frames varying from 3 up to 6 months. Of note, a better outcome regarding migraine frequency in comorbid patients compared to subjects only showing migraine is observed, which may hint to a specific good efficacy in migraineurs with depressive symptoms. However, future studies with longer run time and larger sample sizes are needed to be able to further elaborate on the overall comparability in a meta-analytic setup. All the studies included in our meta-analysis also reported mild and transient side effects and adverse events (typically neck pain, eyelid ptosis, musculoskeletal stiffness, injection site pain, headache). Notably, partly high discontinuation rates were reported in some studies. As stated in the studies, the major reasons why the patients tend to giving up with the treatment are lack of efficacy, lost to follow up, withdrawal of the consent and adverse events. In general, subjects affected by chronic disorders tend to be discontinuous in following treatments which makes the statistical analyses of the experiments less powerful [19, 25].

A limitation of our study derives from the variety of the scales used to assess the impact and severity of migraine and depression, which led to the necessity to perform the statistical analyses on several smaller subgroups although the overall number of subjects is, with more than a thousand individuals, quite large. The division in subgroups weakened the statistical power of our study. This heterogeneity in the scales highlights the aspect that the use of common scales assessing migraine or depression severity would provide a more robust basis for broader and more reliable comparisons. The studies also exhibit a significant variety in their run time, spanning from 3 months to almost 2 years. The duration of treatment is known to have a relevant impact on the treatment success with onabotulinumtoxinA, but we were not able to further assess the impact of this variable because of the limited number of studies in each subgroup. Statistical analyses are also characterized by a very high heterogeneity, but since a very limited amount of studies were included in each analysis, this heterogeneity could not be interpreted as meaningful.

Overall, the onabotulinumtoxinA treatment showed promising results and, in this sense, more studies are needed to eventually confirm the positive effects that have been found in this meta-analysis. It may be possible that some issues related to this specific therapy contribute to the high number of patients giving up on continuing the treatment. This makes more difficult to perform further studies, thus slowing down the progress of the research in this particular area. Nonetheless, we believe that these preliminary results are robust and that further success in the implementation of this therapy will lead to a broader use and to a better health care for chronic migraineurs with depressive symptoms.

## Conclusion

The use of onabotulinumtoxinA in patients with comorbid migraine and major depressive disorder leads to a significant reduction of symptoms of both diseases. OnabotulinumtoxinA may be slightly more effective in patients with both migraine and depression regarding certain disease features (especially for migraine), compared with patients with just one of these two disorders. More studies are needed to assess and compare long time efficacy in chronic migraineurs with depressive symptoms and monomorbid patients.

## Data Availability

All data generated or analyzed during this study are included in this review.

## Abbreviations

MDD: Major depressive disorder
CGRP: calcitonin gene-related peptide
IHS: International headache society
SD: Standard deviation
CI: Confidence interval
HIT6: Headache impact test
PHQ-9: Patient health questionnaire
MIDAS: Migraine disability assessment
BDI: Beck depression inventory
GAD: Generalized anxiety disorder
Zung-D: Zung self-rating depression scale
DASS-21: Depression anxiety stress scale
VAS: Visual analogue scale
